# Laparoscopic Resection of a Left Upper Quadrant Mass Leading to a Surprise Diagnosis

**DOI:** 10.1155/2020/8365061

**Published:** 2020-05-30

**Authors:** Hugo J. R. Bonatti, Reinhardt O. Sahmel, Rodrigo B. Erlich

**Affiliations:** ^1^University of Maryland Community Medical Group, Easton, MD, USA; ^2^Meritus Surgical Specialists, Hagerstown, MD, USA; ^3^University of Maryland Shore Regional Health, Easton, MD, USA; ^4^Dyson Cancer Center, Poughkeepsie, NY, USA

## Abstract

*Background*. Resplenectomy is most commonly done for the treatment of recurrent idiopathic thrombocytopenic purpura (ITP) refractory to medical management due to the regrowth of a missed accessory spleen. *Case Report*. A 66-year-old male had undergone open splenectomy for traumatic rupture 40 years ago. He presented with a leiomyosarcoma of his leg, which was surgically removed. When he developed metastatic disease, chemotherapy was started. He developed left upper quadrant pain, and on CT scan, a 5 cm mass compatible with a sarcoma was found between the tail of the pancreas and the left adrenal gland. During laparoscopy, dense adhesion of the omentum to the abdominal wall and the stomach from his previous splenectomy was divided. The lesser sac was opened through the gastrocolic ligament, and the splenic flexure was taken down. Superior and dorsal to the tail of the pancreas next to the left adrenal gland, the mass was identified and carefully dissected out. The vascular pedicle, which originated from a side branch of the splenic vessels at the tail of the pancreas, was stapled. The gastric fundus showed multiple nodules, and therefore, a modified sleeve gastrectomy was done; also, a 2 cm nodule in segment 5 of the liver and an omental nodule were removed. The tumors and gastrectomy specimen were placed in an endobag and removed through a periumbilical mini-incision. The patient recovered without any complications from the procedure and his LUQ pain resolved. Pathology revealed no sarcoma metastases but accessory spleens in all specimens. *Discussion*. Splenosis with multiple implants within the abdomen after splenectomy for trauma is a rare condition. In our patient, this seems to have been triggered by chemotherapy for his sarcoma resulting in extramedullary hemopoiesis. Laparoscopic removal of accessory spleens can be safely done.

## 1. Introduction

Overall rates of splenectomy have recently declined due to the development of medical therapies for many disorders, embolization of the splenic artery for some disorders (partial splenectomy), and nonoperative management of traumatic splenic laceration [1, 2]. Laparoscopic approach is favored for most cases of elective splenectomy [3], whereas laparotomy is reserved for severe trauma and difficult splenectomies [1, 3, 4]. Laparoscopy is also safe for the removal of accessory spleens [3, 5–7]. Most cases of resplenectomy are done for recurrent idiopathic thrombocytopenic purpura (ITP) refractory to medical management [5–7].

In traumatic splenectomy, the presence of splenules is of benefit and some surgeons implant splenic tissue into the omentum during splenectomy. In case of shattered spleen, splenic tissue dispersed within the abdomen may find access to vascularity and regrow by time. Such regrown spleens may become symptomatic causing pain, bowel obstruction, or hemorrhage [8–11]. CT scan and MRI can be used to locate such regrown spleens, and liver/spleen scan and scan using damaged red blood cells are additional modality to locate the pathology [10]. Several cases of removal of such regrown splenic tissue have been reported, and laparoscopy seems to be feasible [7, 12–14]. If splenic tissue diffusely grows within the abdominal cavity, it is referred to as splenosis and this may be associated with various complications [11, 14].

Significant advances in chemotherapy for sarcomas have recently been made [15]. One of the agents now commonly used is the multitarget tyrosinase inhibitor pazopanib [16], which is usually combined with other cytotoxic drugs. The agent has potent antiangiogenetic properties. A common side effect of chemotherapy is myelotoxicity with cytopenia, and frequently, growth factors such as erythropoietin and filgrastim are administered [17]. These agents not only cause proliferation of bone marrow-based stem cells but also trigger extramedullary hemopoiesis with splenic tissue being one of the targets [18].

## 2. Case Report

**Figure 1:**
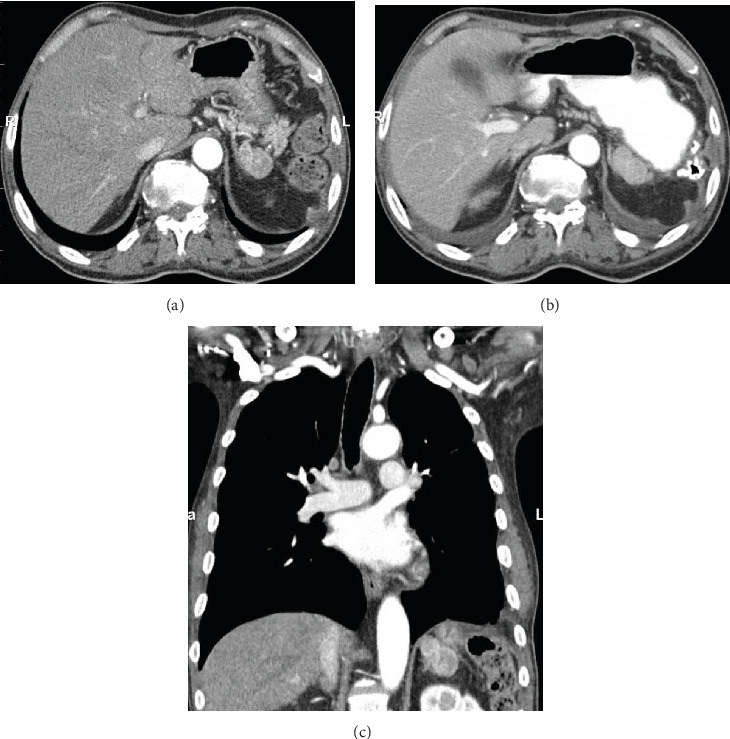
CT scan ((a, b) transverse; (c) coronal view): 5 cm mass close to the tail of the pancreas and next to the left adrenal gland (arrow).

A 66-year-old male had undergone open splenectomy for abdominal blunt trauma 40 years ago. He was well and asymptomatic for almost four decades when he developed a leiomyosarcoma on his leg. This was surgically removed, and he also received radiation to the tumor region. Few months later, metastatic disease was diagnosed and he was started on chemotherapy. Imaging at this time revealed no evidence for intra-abdominal seeds. Chemotherapy with docetaxel/gemcitabine, pazopanib, eribulin, temozolomide, and doxorubicin/olaratumab was initiated, and the patient tolerated the agents well for several months when he started to complain of left upper quadrant (LUQ) pain. On CT scan, a 5 cm mass was found close to the tail of the pancreas and the left adrenal gland (Figures 1(a)–1(c)). This was compatible with a soft tissue sarcoma and thought to be a metastasis of the leiomyosarcoma. As the patient was symptomatic with LUQ pain, decision was made to resect the mass, and the patient was consented for a laparoscopic attempt.

**Figure 2:**
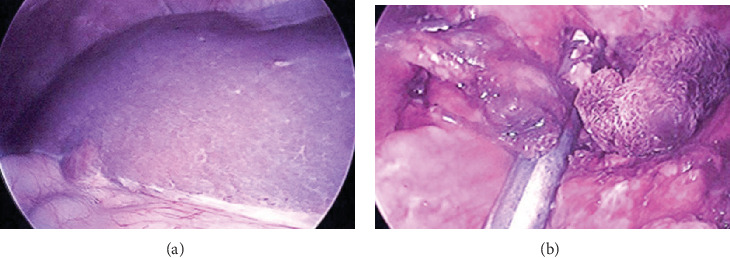
Intraoperative findings. (a) Nodule in segment 5 of the liver. (b) The vascular pedicle of the main nodule is stapled.

In the OR, the patient was placed supine. A 5 mm Fios first entry port in the LUQ was used to establish pneumoperitoneum; another 5 mm port was placed in the epigastrium and a 10-12 mm trocar above the umbilicus using his old laparotomy scar. Dense adhesion of the omentum in the LUQ to the abdominal wall and the stomach was divided. Several nodules were found in the omentum, and another nodule was found in segment 5 of the liver (Figure 2(a)), and it was assumed that these were metastatic leiomyosarcoma lesions. The distal transverse colon was elevated and the lesser sac opened through the gastrocolic ligament using the harmonic scalpel. The splenic flexure was taken down, and the stomach was lifted anteriorly and medially to expose the tail of the pancreas. At the fundus, multiple additional nodules were found. The left adrenal gland was exposed, and just lateral to it, the 5 cm mass was found. It was carefully dissected out with a safety margin. Dorsally, it was lifted off the left kidney. Blood supply for the mass originated from a side branch of the splenic artery and splenic vein at the tail of the pancreas. An Echelon stapler with a vascular load was used to divide the blood vessels (Figure 2(b)). Next, the largest omental nodule was resected, and thereafter, a liver wedge resection in segment 5 including the previously described nodule was performed. The primary lesion, the liver wedge, and the omental nodule were placed into a retrieval bag. Finally, a limited gastric sleeve resection including the nodules at the fundus was performed, and the gastrectomy specimen was placed into another retrieval bag. The specimens were removed from the abdomen through a 3 cm periumbilical incision including the 10-12 mm port site.

**Figure 3:**
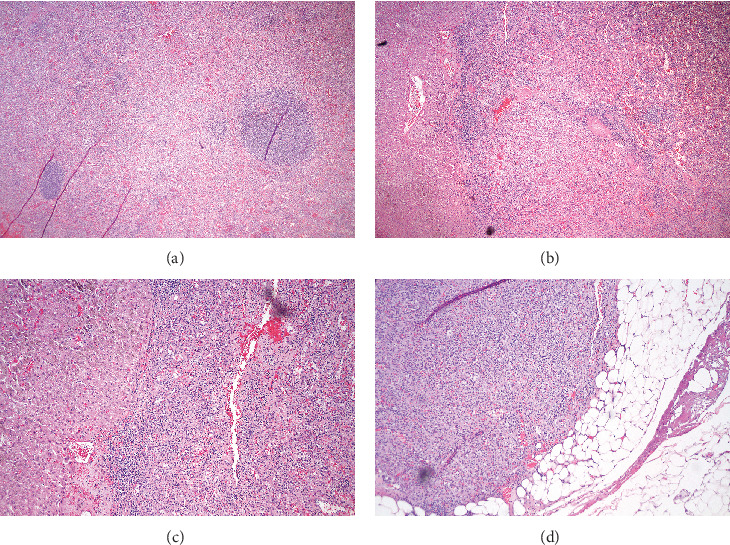
Histopathology shows splenic tissue in the (a) main nodule, (b) liver, (c) gastric fundus, and (d) omentum.

The patient recovered well from the procedure without any complications and was discharged on day 3. The LUQ pain had resolved. Pathology revealed splenic tissue in all four specimens and no evidence for leiomyosarcoma metastases (Figures 3(a)–3(d)). The patient is well and alive with a stable platelet count, no LUQ pain, and no progression of metastatic leiomyosarcoma after 24 months continuing chemotherapy.

## 3. Discussion

We present a patient with metastatic leiomyosarcoma with a new painful mass in the LUQ. On laparoscopy, multiple additional intra-abdominal lesions were found, and it was assumed that diffuse spread of the malignancy was present. To our surprise, on pathology, the masses did not reveal metastatic leiomyosarcoma but accessory spleens reflecting a diagnosis of splenosis.

On a CT scan one year earlier, this condition was not present, and the LUQ nodule developed and became symptomatic only after chemotherapy including pazopanib had been initiated. The agent is used to treat various types of malignancies and has shown promising features in the treatment of soft tissue sarcoma including metastatic leiomyosarcoma [15], but also, other new drugs are available. A common side effect of chemotherapy is pancytopenia, and our patient was receiving hematopoietic growth factors including erythropoietin and granulocyte stimulating factor. Despite the growing splenic tissue, he did not develop thrombocytopenia. We believe polychemotherapy and the growth factors triggered extramedullary hemopoiesis causing the spilled splenic tissue from his trauma 40 years earlier to proliferate.

Such a condition had not been taken in consideration by our team, and we assumed metastatic leiomyosarcoma when the patient presented with LUQ pain and the lesion was found on CT scan. If an accessory spleen had been considered, a liver/spleen scan may have been beneficial prior to surgery [10]. Surgical removal was indicated due to the pain; also, reduction of tumor burden would be achieved, and it was also discussed that fresh tissue would allow optimization of therapy based on genetic analysis. Despite the previous open splenectomy, a laparoscopic approach was chosen. Our case confirms that such complex laparoscopic surgery in the left upper quadrant can be done even in patients with a history of splenectomy including open surgery for trauma. We also confirm that laparoscopic resplenectomy can be safely done in patients who develop symptoms associated with a regrown accessory spleen [3, 5–7, 10, 19–27]. The procedure may be technically challenging, and extensive lysis of adhesions may be required.

Regrowth of splenic tissue causing problems is most commonly associated with recurrent ITP, and most patients are asymptomatic except for their thrombocytopenia [28]. Splenosis is different as it may cause abdominal pain and other severe complications such as bowel perforation, obstruction, and strangulation [8, 9, 11–14, 29]. It may be a diagnostic challenge especially in oncologic patients [29, 30]. Splenosis may be due to accessory spleens unrecognized during workup and during splenectomy. In addition, the condition may be due to implantation of splenic tissue during trauma or surgery if splenic tissue is spilled. In our case, we assume that the spillage of splenic tissue occurred during his trauma causing development of multiple accessory spleens. The blood supply of the largest accessory spleen originated from the splenic vessels in the area of the tail of the pancreas, and we secured the pedicle with a stapler. The fundus with the multiple nodules was resected using a modified sleeve gastrectomy technique. The postoperative course was uneventful and the patient's LUQ pain resolved, and he is well after 24 months.

Laparoscopy should be the preferred approach to explore pathologies in the left upper quadrant even in patients with a history of open splenectomy. The case also emphasizes that resplenectomy can be safely done using the laparoscopic approach. It should be noted that some chemotherapies and administration of hematopoietic growth factors may trigger hypertrophy of splenic tissue spilled within the abdomen as well as growth of accessory spleens. This pathology should be considered, and special imaging such as technetium-99m (Tc-99m) sulfur colloid scintigraphy or Tc-99m heat-damaged red blood cell scintigraphy may be advisable in similar cases [31]. These tests are commonly used to find accessory spleens in patients with recurrent ITP after splenectomy, and Short et al. reported that Tc-99m sulfur colloid single-positron emission computed tomography (SPECT) was used in a patient to distinguish splenosis from diffuse intra-abdominal metastatic disease suspected on CT scan [31].
